# Plastic changes in primate motor cortex following paired peripheral nerve stimulation

**DOI:** 10.1152/jn.00288.2020

**Published:** 2020-12-02

**Authors:** Bonne Habekost, Maria Germann, Stuart N. Baker

**Affiliations:** Faculty of Medical Sciences, Newcastle University, Newcastle upon Tyne, United Kingdom

**Keywords:** long-term depression, long-term potentiation, motor cortex, plasticity

## Abstract

Repeated paired stimulation of two peripheral nerves can produce lasting changes in motor cortical excitability, but little is known of the underlying neuronal basis. Here, we trained two macaque monkeys to perform selective thumb and index finger abduction movements. Neural activity was recorded from the contralateral primary motor cortex during task performance, and following stimulation of the ulnar and median nerves, and the nerve supplying the extensor digitorum communis (EDC) muscle. Responses were compared before and after 1 h of synchronous or asynchronous paired ulnar/median nerve stimulation. Task performance was significantly enhanced after asynchronous and impaired after synchronous stimulation. The amplitude of short latency neural responses to median and ulnar nerve stimulation was increased after asynchronous stimulation; later components were reduced after synchronous stimulation. Synchronous stimulation increased neural activity during thumb movement and decreased it during index finger movement; asynchronous stimulation decreased activity during both movements. To assess how well neural activity could separate behavioral or sensory conditions, linear discriminant analysis was used to decode which nerve was stimulated, or which digit moved. Decoding accuracy for nerve stimulation was decreased after synchronous and increased after asynchronous paired stimulation. Decoding accuracy for task performance was decreased after synchronous but was unchanged after asynchronous paired stimulation. Paired stimulation produces changes in motor cortical circuits that outlast the stimulation. Some of these changes depend on precise stimulus timing.

**NEW & NOTEWORTHY** Paired stimulation of peripheral nerves for 1 h induced lasting changes in neural responses within the motor cortex to nerve stimulation and to performance of a behavioral task. These changes were sufficient to alter the efficiency with which activity could encode stimulus type. Stimuli that can be easily applied noninvasively in human subjects can alter central motor circuits.

## Graphical abstract

**Figure:**
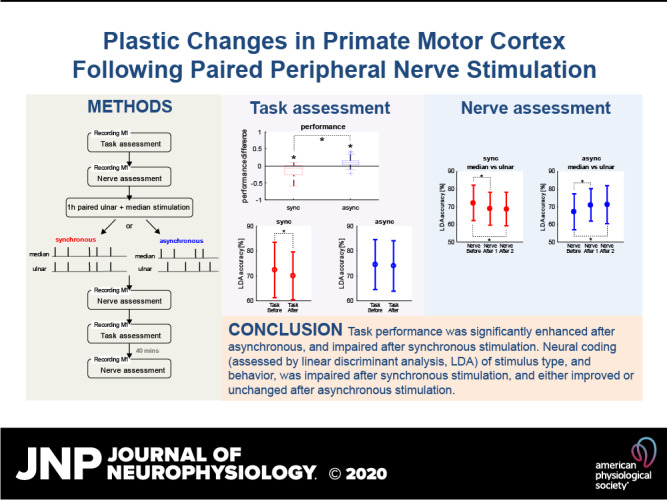

## INTRODUCTION

Synaptic connections within the motor systems are not fixed but can be modified based on experience. Such plastic changes are likely to underlie motor learning (1, 2). A wide variety of neuromodulation strategies have been developed to exploit these natural mechanisms in humans to induce long-term plastic changes in motor output. This could enhance recovery following damage to the motor system, such as after stroke or spinal cord injury, although to date these methods have not entered routine clinical practice (3). Often, the available protocols involve noninvasive stimulation of the central nervous system using transcranial magnetic brain stimulation (TMS). Plasticity can be induced by repetitive TMS (4), or by pairing TMS with a peripheral stimulus (5–7), with natural activity generated during voluntary movements (8–10) or with motor imagery (11, 12). These different approaches each have advantages and disadvantages, for example, in their relative ability to produce plastic changes in different muscle groups (13). However, all share the disadvantage of requiring a bulky and expensive TMS machine and precise placement of the stimulating coil on the scalp. This limits their applicability to a laboratory setting, and the number of stimuli is constrained to what is feasible during a laboratory visit. Larger plastic changes might be generated by more stimuli (e.g., 14); this therefore could be a critical constraint on applying such approaches to clinical rehabilitation.

An alternative strategy to induce plasticity is to exploit natural inputs to activate motor pathways trans-synaptically. Examples would be stimulation of a peripheral nerve to activate somatosensory inputs to motor cortex (15, 16) or a loud click to stimulate the vestibular system (17, 18). Stimulation of the motor point of two muscles (19–22), of two peripheral nerves (23–26), or of the motor point of a muscle paired with a click (27) can all generate plastic changes in motor output. We recently developed a portable device capable of delivering such stimuli while a subject engages in their usual daily activities and showed that this could induce plastic changes in healthy individuals (27–29) and in stroke patients (30). This opens up the possibility of taking plasticity protocols outside the laboratory, where large numbers of stimuli can be delivered to generate clinically significant effects.

Given this promise, it is important to understand the underlying mechanisms by which noninvasive paired stimulation protocols generate changes in output. To date, all studies of these approaches have used noninvasive measurements in human subjects to assess motor plasticity. Synchronous (associative) stimulation of two afferent inputs generates an increase in motor-evoked potential (MEP) from the primary motor cortex (M1) (19, 21, 22, 24, 26) and an increased overlap of muscle representations within M1 assessed by TMS mapping (22, 26). By contrast, stimulating two afferent inputs alternately (nonassociatively) does not produce excitability changes in the sensorimotor cortex (21, 22). In patients with focal hand dystonia, nonassociative stimulation reduces the area of the representation of a stimulated muscle within M1 and increases the separation of muscles in the cortical map (31–33). Plastic changes in muscle responses to stimulation of M1 after associative afferent stimulation seem to occur mainly in M1, as there is no change in motoneuron excitability as assessed by F waves (26). Intracortical facilitation (ICF, 34) increases after paired associative afferent stimulation (20), which may also support the idea that plastic changes occur in the motor cortex, although a partial subcortical contribution to ICF has been proposed (35).

Plasticity can also be induced in the somatosensory system by paired associative stimulation of tactile afferents. This can produce improvements in a spatial discrimination task (36–38) and modify the somatosensory-evoked potential (38). These observations are underpinned by direct measurements of cortical activity in animals, which reveal increased firing of neurons in the primary somatosensory cortex after paired stimulation (37). Unfortunately, no studies to date have examined the effect of paired afferent stimuli on neural recordings from the motor system.

In this paper, we report direct recordings of neural activity in M1 from awake behaving monkeys. We examined the modulation of activity during performance of a behavioral task requiring fine fractionated finger movements, as well as after electrical stimulation of peripheral afferents. Paired afferent stimulation was then delivered, and changes in neural responses assessed. We found that short-latency responses to afferent input, and the modulation of activity with behavior, were changed after a period of paired stimulation. Changes were seen after both synchronous and asynchronous stimulation, although the details of these changes differed. Neural coding of stimulus type, and behavior, was impaired after synchronous stimulation, and either improved or unchanged after asynchronous stimulation. This work reveals, for the first time, the fine-scale changes occurring within M1 after a plasticity protocol, which can be applied noninvasively in humans.

## METHODS

### Behavioral Task

**Figure 1.**
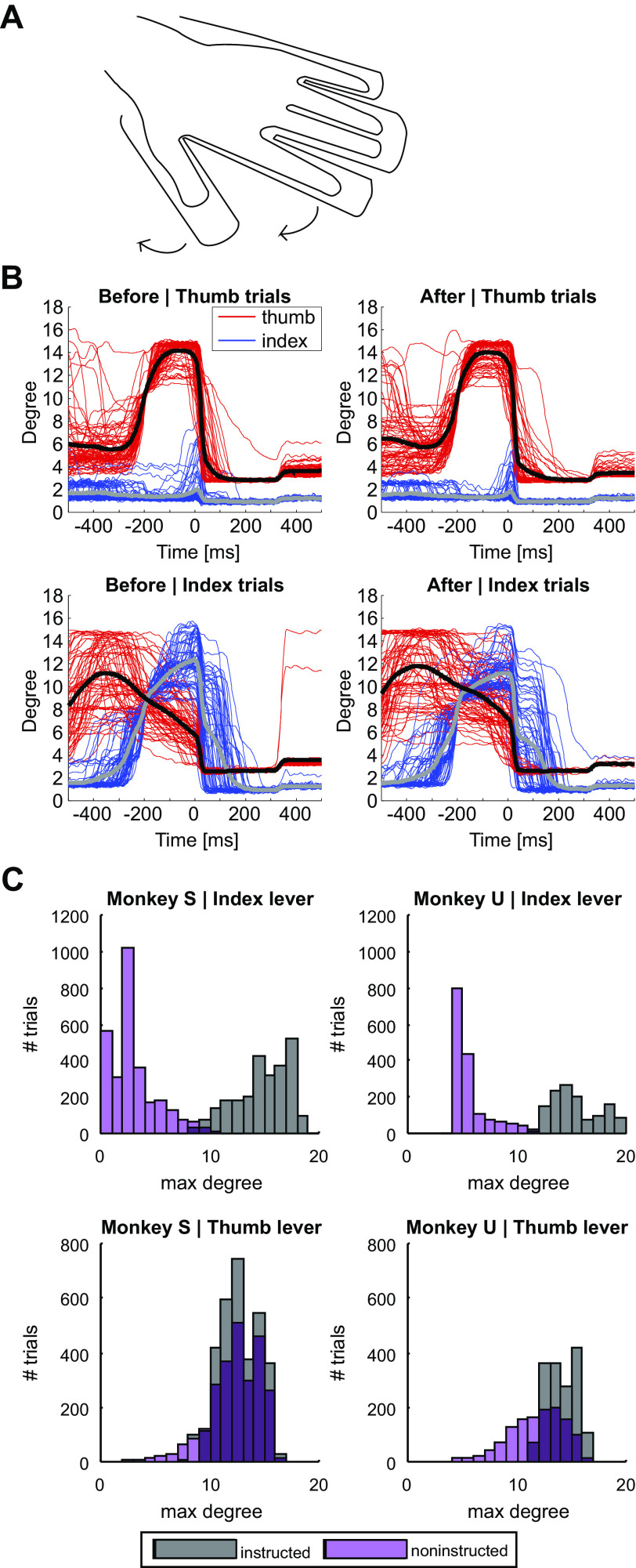
Finger abduction task. *A*: monkeys were conditioned to position their hand in a custom-made manipulandum. The movement of either the thumb or the index finger was then instructed by a vibrational cue delivered through miniature motors underneath each plastic shaft of the manipulandum. *B*: lever positions for each trial (*n* = 282) during one example session for thumb (red) and index finger (blue) before (*left*) and after (*right*) synchronous stimulation. Average lever positions are depicted as black (for thumb) and gray (for index finger) lines. For trials requiring thumb abduction (*top*), monkeys were able to move their thumb very selectively, whereas for trials requiring index finger abduction (*bottom*) the noninstructed digit moved more extensively. *C*: maximum degree of displacement in each trial (*n* = 5,833 trials Monkey S, *n* = 2,875 trials Monkey U) for both levers during instructed (gray) and noninstructed (purple) trials for monkey S (*left*) and monkey U (*right*) during baseline task assessment.

The study used two female *Macaca mulatta* monkeys (monkey S: 6 yr old, weight 6.30 kg; monkey U: 4 yr old, weight 4.76 kg). All experimental procedures were carried out under appropriate licenses from the UK Home Office and were approved by the Animal Welfare and Ethical Review Board of Newcastle University. The monkeys were trained to place the left hand in a manipulandum, which contained separate channels for the thumb, index finger, and *digit 5* and a single well for *digits 3* and *4*. The thumb and index finger channels were pivoted coaxial with the relevant metacarpophalangeal joint, constraining movement to the direction of digit abduction (Fig. 1*A*). Angular displacement was sensed by optical encoders (ENX10 EASY 1024IMP, Maxon Motors, Sachseln, Switzerland) and opposed by miniature torque motors (DCX10L with GPX10 64:1 planetary gearhead, also Maxon Motors) placed on the shaft. Current in the motor was controlled continually by a computer program as a function of the displacement to simulate the action of a spring (initial torque 28 mNm, spring constant 0.79 mNm/°). A miniature vibrator (Cat. No.: 308-100, Precision Microdrives Ltd, London, UK) was placed under the thumb and index finger. When both digits had been held below a resting threshold for 2 s, activation of one of these vibrators for 200 ms cued which digit was to be moved. The monkey was then required to abduct the instructed digit more than a threshold level from the resting position, whilst keeping the noninstructed digit below a maximal-motion threshold. Both thresholds were gradually refined during the training process, until the monkey was capable of selective digit movement. During thumb-instructed trials, final thresholds were set such that the index finger had to stay below 11°, while the thumb had to exceed 7°. During index-instructed trials, the thumb had to stay below 17°, while the index finger had to exceed 8°. After a successful trial, motor torque was raised to 56 mNm to return the digits to the resting position. Different auditory cues indicated a successful or failed trial. Successful task performance earnt a food reward.

### Surgical Preparation

Once behavioral training was complete, each monkey underwent an implant surgery under full aseptic conditions and deep general anesthesia. Anesthesia was induced by 10 mg · kg^−1^ ketamine (IM) and maintained by inhalation of sevoflurane (2.0–3.5% in 100% O_2_), supplemented with a continuous intravenous infusion of alfentanil (0.025 mg · kg^−1^ · h^−1^). A full program of postoperative analgesia (6 mg · kg^−1^ meloxicam) and antibiotic (6 mg · kg^−1^ ceftiofur) was given. During this surgery, two custom-made bipolar nerve cuff electrodes were placed on the median and ulnar nerves in the upper arm. The nerves were implanted at the same proximodistal location in the arm, so that the conduction time for an afferent volley to reach the central nervous system after stimulation of each cuff should be similar. The median nerve innervates most forearm flexors and the intrinsic hand muscles in the thenar eminence, together with a cutaneous field on the palmar surface of the radial side of the hand and digits. The ulnar nerve innervates the flexor carpi ulnaris in the forearm, the remaining intrinsic hand muscles, and the rest of the skin on the palmar side. No cuff electrode was placed around the radial nerve, which innervates forearm extensors and skin on the dorsal aspect of the hand. However, a pair of Teflon-insulated stainless steel wires were inserted into the extensor digitorum communis (EDC) muscle, close to the motor point, for stimulation of afferents from this muscle, which is supplied by the radial nerve. A headpiece made of carbon fiber-reinforced plastic (Kentron CA30 PEEK, Engineering & Design Plastics Ltd, Cambridge, UK) was fixed to the skull with expanding bolt assemblies, allowing atraumatic head fixation (39). Wires from the EMG and nerve cuff implants were routed subcutaneously to connectors fixed to the headpiece. A recording chamber was positioned over the hand area of the right primary motor cortex (M1); chamber placement was determined based on structural magnetic resonance imaging (MRI) scans gathered some months before the surgery. In a second brief surgery, two fine tungsten electrodes (LF501G, Microprobes Inc) were implanted in the pyramidal tract at the medulla. Electrodes were positioned using the double angle stereotaxic technique described in Ref. 40 and fixed at the depth with the lowest threshold to evoke an antidromic field potential in recordings from the dura overlying M1.

### Neural Recordings

After recovery from implant surgeries was complete, recording sessions commenced. The nerve cuff and EDC electrodes were first stimulated with the monkey at rest to determine the motor threshold (MT). Stimulation used isolated, constant current biphasic pulses (1 ms per phase; either Model 2100, AM Systems Inc, Sequim, WA, or DS4, Digitimer Ltd, Welwyn Garden City, UK). Motor thresholds for EDC were 1.2 or 2.2 mA; for the nerve cuffs, they varied between 400 and 600 μA. Subsequent stimulation used an intensity 2× MT, which should activate *group I* and *II* muscle afferents (41) as well as fast-conducting cutaneous fibers (42).

Experimental sessions were performed three times per week. An Eckhorn microdrive (43) loaded with glass-insulated platinum microelectrodes (tip impedance 1–2 MΩ) was used to make recordings of neural activity from M1 through the chamber. We searched for identified pyramidal tract neurons (PTNs), which were activated at fixed latency from the pyramidal tract electrodes (biphasic pulses, 0.2 ms per phase, up to 400 μA); the antidromic nature of the activation was confirmed by a collision test (44). When recording from PTNs it is essential that the single unit is well isolated, so that changes in firing can be definitively assigned to the corticospinal system. In many cases, we found that subtle shifts in recordings throughout the lengthy protocol meant that this high standard could not be reached, and some contamination from other nearby units was likely. Accordingly, in these cases, we analyzed multiunit activity (MUA, see *Extracting Neural Activity*). At the end of some recording sessions, intracortical microstimulation was delivered through the microelectrodes (13 pulses, interpulse interval 3 ms) and the nature and threshold of any evoked movements noted.

### Experimental Design and Stimulation Sequences

**Figure 2.**
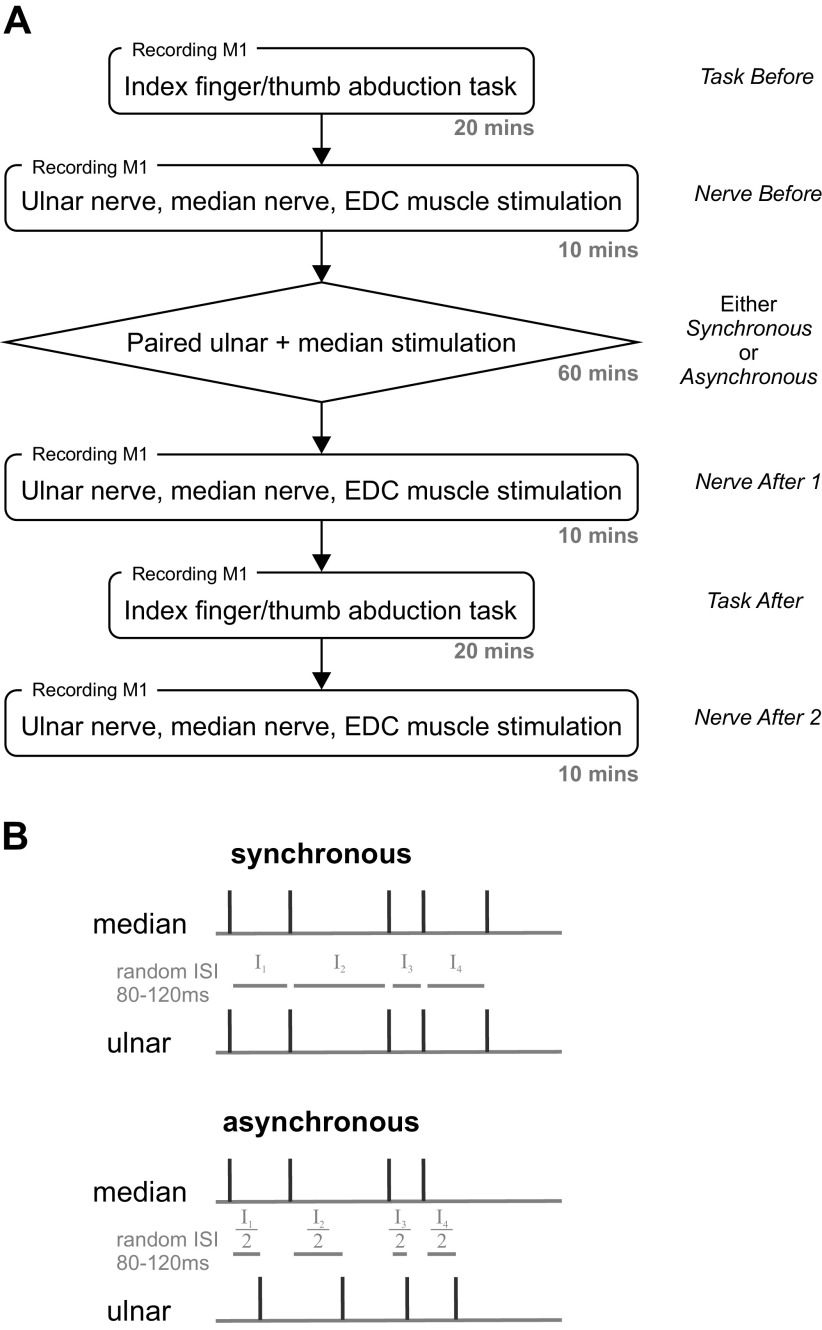
Experimental design. *A*: experimental flow with approximate length of time for each part and designations used in the text. *B*: stimulation protocols for synchronous (*top*) and asynchronous (*bottom*) paired median and ulnar nerve stimulation. Note that for synchronous stimulation, the two nerves were stimulated at the same time. EDC, extensor digitorum communis; ISI, interstimulus interval.

Once single- or multiunit activity had been located, the experiment had six parts (Fig. 2*A*), during which neural activity was recorded, as follows:

*Task before.* The behavioral task was performed for 150 successful trials. On average this part took 20.1 ± 7.7 min (means ± SD).

*Nerve before.* The EDC, median, and ulnar nerve electrodes were stimulated individually, with the EDC serving as a control. The nerves were stimulated in a randomized order with interstimulus interval 500 ms; 300 stimuli of each type were given while the monkey sat quietly. Stimuli were delivered by a single isolated stimulator (as under *Neural Recordings*), which was switched to the different electrodes under computer control using a custom relay circuit. Relays were switched 10 ms before the stimulus to allow time for the contacts to settle before current was passed. On average this part lasted 10.7 ± 2.7 min.

*Paired stimulation (intervention).* The median and ulnar nerves were either stimulated synchronously or asynchronously for around 1 h while the monkey rested quietly, following a similar protocol as previously used in humans (21, 22). For synchronous stimulation, the two nerves were activated at the same time, with interstimulus interval random chosen from 80 to 120 ms (uniform distribution). For asynchronous stimulation, one nerve was stimulated at the start of the interstimulus interval chosen for that trial, and the other was stimulated halfway through the interval. The two stimulation protocols are depicted in Fig. 2*B*. On average, paired stimulation lasted 61.1 ± 0.8 min, the exact duration varied due to the randomized intervals.

*Nerve after 1.* The individual stimulation of EDC and nerve electrodes was repeated, as for the assessment before intervention. On average, this part lasted 10.6 ± 2.4 min.

*Task after.* The behavioral task was performed for 150 further trials. On average, this part took 26.2 ± 10.6 min.

*Nerve after 2.* A further round of individual stimulation was recorded. This was performed immediately after the task performance, and was 41 ± 13 min after the end of the paired stimulation. On average, this part lasted 10.5 ± 2.4 min.

Overall, neural activity was recorded for a total of 139 ± 24 min. Note that *task before* was performed before *nerve before* in the baseline assessment, but *task after* was performed after *nerve after 1* after intervention. This meant that *task after* was recorded on average 10.6 ± 2.4 min after the end of the intervention. Synchronous and asynchronous interventions were applied alternatingly from one experimental session to the next; sessions were interleaved by at least 36 h to avoid potentially long-lasting effects influencing the next session.

Recordings of task lever signals (sampling rate 1 kHz), neural activity (bandpass 300 Hz–10 kHz; sampling rate 25 kHz), and digital markers for task events and stimuli were captured to hard disk with high speed data acquisition cards (National Instruments, Austin, TX). Stimulus timing was controlled by Spike2 software and a micro1401 interface (Cambridge Electronic Design, Cambridge, UK).

### Behavioral Analysis

During an epoch assessing behavioral task performance, we counted the number of failed trials (when the noninstructed digit moved beyond the allowed threshold) as *e*.

The performance *A* was expressed as:

$$A=\;\left(\frac{\text{c}}{\text{e}+c}\right)$$

where *c* is the number of correct trials corresponding to error trial numbers *e*. The speed *s* was defined as

$$s=\frac{c}{t}$$

where *t* is the duration of the recording in which *c* correct trials were performed.

Differences in raw error counts *e*, performance *A*, and speed *s* before and after the intervention were computed and compared across sessions for the synchronous and asynchronous stimulation. Separate measures were calculated for trials with the index or thumb as the instructed digit and for total performance in which both trial types were combined. Two-way ANOVAs were performed to determine the effect of MONKEY (monkey S, monkey U) and STIMULATION (synchronous, asynchronous) on performance, speed, and total number of errors. Post hoc statistical comparisons of parameters before and after the intervention were performed by *t* tests. The Benjamini–Hochberg procedure (45) was used to correct for multiple comparisons with an overall false discovery rate of 0.05. In the results section, uncorrected *P* values are quoted, but these are only stated to be significant if they met the Benjamini–Hochberg criterion.

### Extracting Neural Activity

Spike events for MUA were extracted from the raw waveform recordings based on crossing a threshold ± θ, where θ was determined by Quiroga et al. (46):

$$\text{θ}=5\times \text{median}\left\{\frac{\left|x\right|}{0.6745}\right\}$$

Here |*x*| is the absolute value of the waveform, and the median is taken over the entire available recording. Spike events for identified PTNs were extracted using custom scripts, which allowed the threshold to be adjusted from the automatically determined value to improve isolation. Subsequent separation of wave shapes used cluster cutting on parameters determined from principal components analysis (47). Only clean single units with stable spike waveform shapes and no interspike intervals < 1 ms were used for analysis of PTN firing.

Only MUAs that showed task modulation were used in this paper. The peak modulation was measured from the perievent time histogram (PETH) as the difference between the maximal and minimal rate, over the period from 500 ms before to 200 ms after the completion of a successful trial (10-ms bin width). A shuffling method determined whether the modulation was significantly different from zero, as used in previous work from this laboratory (48). Interspike intervals for each trial were shuffled randomly 1,000 times; for each shuffle, the PETH was recalculated, and the peak modulation measured. If the modulation of the unshuffled PETH was >95% of the modulations after shuffling, the cell was assumed to be significantly modulated by that event (*P* < 0.05).

### Analysis

Peristimulus time histograms (PSTHs) of neural activity were constructed relative to stimulation of the EDC, median, and ulnar nerves (window 20 ms before to 100 ms after the stimulus; bin width 1 ms). For responses to nerve stimulation, the bins from 0 ms before to 6 ms after the stimulus were blanked (set to zero counts) to avoid contamination by the stimulus artefact. In some recordings, the relay switching 10 ms before the stimulus (see *Experimental Design and Stimulation Sequences*) also generated a small artifact; we accordingly also blanked this bin to avoid artifactual contamination.

The change in PSTH response evoked by a particular nerve stimulation was determined for each bin by calculating a *Z*-score as:

$$Z=\frac{\left(\frac{N_{aft}}{m_{aft}}-\frac{B_{aft}}{m_{aft}\;n_{aft}}\right)-\;\left(\frac{N_{bef}}{m_{bef}}-\frac{B_{bef}}{m_{bef}\;n_{bef}}\right)}{\sqrt{\frac{N_{aft}}{m_{aft}{}^{2}}+\frac{B_{aft}}{m_{aft}{}^{2}\;n_{aft}{}^{2}}+\frac{N_{bef}}{m_{bef}{}^{2}}+\frac{B_{bef}}{m_{bef}{}^{2}\;n_{bef}{}^{2}}}}$$

where *N* is the number of spikes in this 1 ms-wide bin, *m* is the number of trials, *B* is the number of spikes in the baseline period, *n* is the number of bins of the baseline period, and the subscripts relate to measurements taken before (bef) and after (aft) the intervention. For the task assessment, perievent time histograms were constructed relative to the completion of successful trials of the task (window 500 ms before to 200 ms after; bin width 10 ms). Trials were counted as successful if both digits were held below a resting threshold for 2 s, followed by the instructed digit abducting more than the minimal-motion threshold while the noninstructed digit stayed below maximal-motion threshold. As there was no clear baseline period, change in PETH response was determined for each 10-ms-wide bin by calculating the *Z*-score as:

$$Z=\;\frac{\left(\frac{N_{aft}}{m_{aft}}\right)-\;\left(\frac{N_{bef}}{m_{bef}}\right)}{\sqrt{\frac{N_{aft}}{m_{aft}{}^{2}}+\frac{N_{bef}}{m_{bef}{}^{2}}}}$$

For PSTHs and PETHs, average firing rate across MUAs were compared between synchronous and asynchronous intervention with two-sample *t* tests, with a significance level of *P* < 0.05 adjusted for multiple comparisons by the Benjamini–Hochberg procedure.

The above formulae assume that spike counts will follow a Poisson distribution where the variance equals the mean and is based on an approach used by previous studies (49, 50). On the null hypothesis that the intervention does not change the neural response, *Z* should be normally distributed with mean zero and standard deviation one.

A measure of the difference averaged across the population was calculated as:

$$\bar{Z}=\;\frac{1}{\sqrt{N}}\sum_{i=1}^{N}Z_{i}$$

where *N* is the total number of cells. Normalizing by the factor of $\sqrt{N}$ ensures that $\bar{Z}$ will also have zero mean and unit standard deviation on the null hypothesis that the neural response is unaffected by the intervention.

The *Z*-score analysis provided a fine-grained display of how the modulation of neural activity during task performance or after nerve stimulation changed after the paired stimulation intervention. To complement this, we also performed a linear discriminant analysis (LDA; 51). This measured how effectively trials could be classified on the basis of the neural firing pattern; LDA yielded a single summary measure of how different neural firing was in two conditions. Single trial data containing the times of detected neural activity were converted to estimates of instantaneous firing rate by convolution with a Gaussian kernel (52) with the same bin widths and time windows used for the PSTHs. All available trials except one were used to estimate the optimal linear classifier (MATLAB function fitcdiscr); this classifier then predicted the class of the excluded trial. The process was repeated with each trial left out in turn; comparison of the actual with the predicted class allowed calculation of the LDA accuracy. LDA was always performed pairwise, e.g., to compare behavioral trials when index finger versus thumb was instructed, or when ulnar versus median nerve was stimulated; this meant that the chance classification rate expected if neural activity did not differ between the compared conditions was 50%. Across a population of recordings, Wilcoxon signed-rank tests were used to compare LDA accuracy before and after intervention, with a significance level of *P* < 0.05 adjusted for multiple comparisons by the Benjamini–Hochberg procedure. Monte Carlo methods were also performed to determine if changes in LDA accuracy were more than expected by chance for an individual MUA or PTN.

All data analysis and visualization was performed with custom-written scripts in the MATLAB environment (MathWorks Inc, Natick, MA).

## RESULTS

### Task Performance

Figure 1*B* illustrates example lever displacement data from one session. For both monkeys, there was typically an asymmetry in selectivity: the index finger was moved very little during thumb-instructed trials, whereas the thumb was moved (but to a smaller extent) during index-instructed trials. This asymmetry can be seen across all sessions, as shown in Fig. 1*C* (measured from the baseline period *task before*, Fig. 2*A*). Displacements of the index finger during noninstructed and instructed trials were cleanly separated, while thumb displacements overlapped between noninstructed and instructed trials.

### Influence of Paired Nerve Stimulation on Task Performance

**Figure 3.**
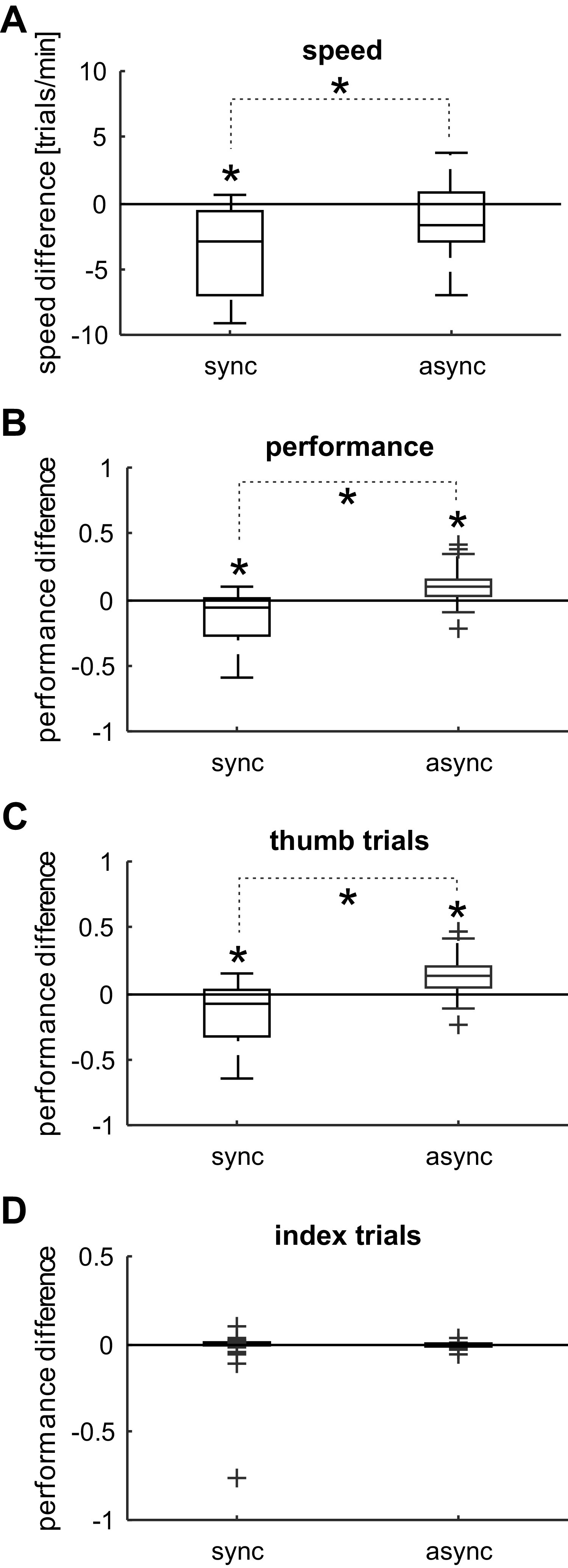
Box and whisker plots for speed and performance differences after synchronous (*n *= 25 sessions) and asynchronous (*n* = 22 sessions) stimulation. Note that values > 0 indicate better, < 0 worse performance after the intervention. Significant differences between the stimulation protocols are indicated with an asterisk on a dotted line (*P* < 0.05, two-sample *t* test, adjusted with the Benjamini–Hochberg procedure). Significant changes after a stimulation protocol are indicated with an asterisk (*P* < 0.05, one-sample *t* test, adjusted with the Benjamini–Hochberg procedure). *A*: speed difference after synchronous (sync) and asynchronous (async) stimulation. *B*: overall performance difference after synchronous and asynchronous stimulation. *C*: performance difference for thumb instructed trials after synchronous and asynchronous stimulation. *D*: performance difference for index instructed trials after synchronous and asynchronous stimulation. Due to the small number of errors made with the noninstructed digit, this box plot collapsed into the *x*-axis.

Figure 3 presents data on how the behavioral performance on the task changed after paired stimulation (comparing *task before* with *task after*, see Fig. 2*A*). Figure 3*A* shows the change in speed *s* (number of correct trials per minute) after synchronous and asynchronous stimulation. ANOVA showed no effect of Monkey (*F*_1,43_ = 2.84, *P* = 0.099) but a significant effect of Stimulation (*F*_1,43_ = 9.18, *P* = 0.004) and their interaction (*F*_1,43 _= 4.31, *P* = 0.0439) on speed. Post hoc *t* tests revealed that monkeys were significantly slower after the synchronous intervention (*P* = 7.51 × 10^−6^) but not the asynchronous intervention (*P* = 0.0393, not significant after correcting for multiple comparisons). Also, the change in speed *s* was significantly different between the synchronous and asynchronous intervention (*P* = 0.0113), with monkeys performing significantly slower after synchronous compared to asynchronous intervention.

Figure 3*B* shows the change of performance *A*, defined as the fraction of trials that were successful (see methods) after synchronous and asynchronous stimulation. ANOVA showed no effect of Monkey (*F*_1,43_ = 0.55, *P* = 0.463), but a significant effect of Stimulation (*F*_1,43_ = 29.38, *P* < 0.001) and their interaction (*F*_1,43_ = 8.37, *P* = 0.006) on performance measure *A*. Post hoc *t* tests revealed that performance was significantly reduced after the synchronous intervention (*P* = 0.0012) and significantly increased after asynchronous intervention (*P* = 0.0047). The difference in performance change between the synchronous and asynchronous interventions was also significant (*P* = 2.00 × 10^−5^).

Figure 3, *C* and *D*, compares the performance *A* before and after synchronous and asynchronous stimulation for each trial type. ANOVA on the performance during thumb-instructed trials (Fig. 3*C*) showed no effect of MONKEY (*F*_1,43 _= 1.76, *P* = 0.192) but a significant effect of STIMULATION (*F*_1,43 _= 31.73, *P* < 0.0001) and their interaction (*F*_1,43_ = 11.77, *P* = 0.0013). Post hoc *t* tests revealed that performance during thumb instructed trials was significantly reduced after the synchronous intervention (*P* = 0.0037) and significantly increased after asynchronous intervention (*P* = 0.0047). The difference in performance change between the synchronous and asynchronous interventions was also significant (*P* = 2.58 × 10^−5^).

ANOVA on the performance measure *A* during index instructed trials (Fig. 3*D*) showed no effect of Monkey (*F*_1,43_ = 1.76, *P* = 0.192), nor of Stimulation (*F*_1,43_ = 31.73, *P* < 0.0001), or their interaction (*F*_1,43_ = 11.77, *P* = 0.0013). It should be stressed that the number of errors made with the thumb (during index instructed trials) was much smaller compared with the number of errors made with the index (during thumb-instructed trials), and the average performance difference for index instructed trials did not significantly differ from 0. This is not surprising, given that the threshold window for the thumb lever allowed less selective movements.

### Neuronal Activity Database

**Figure 4.**
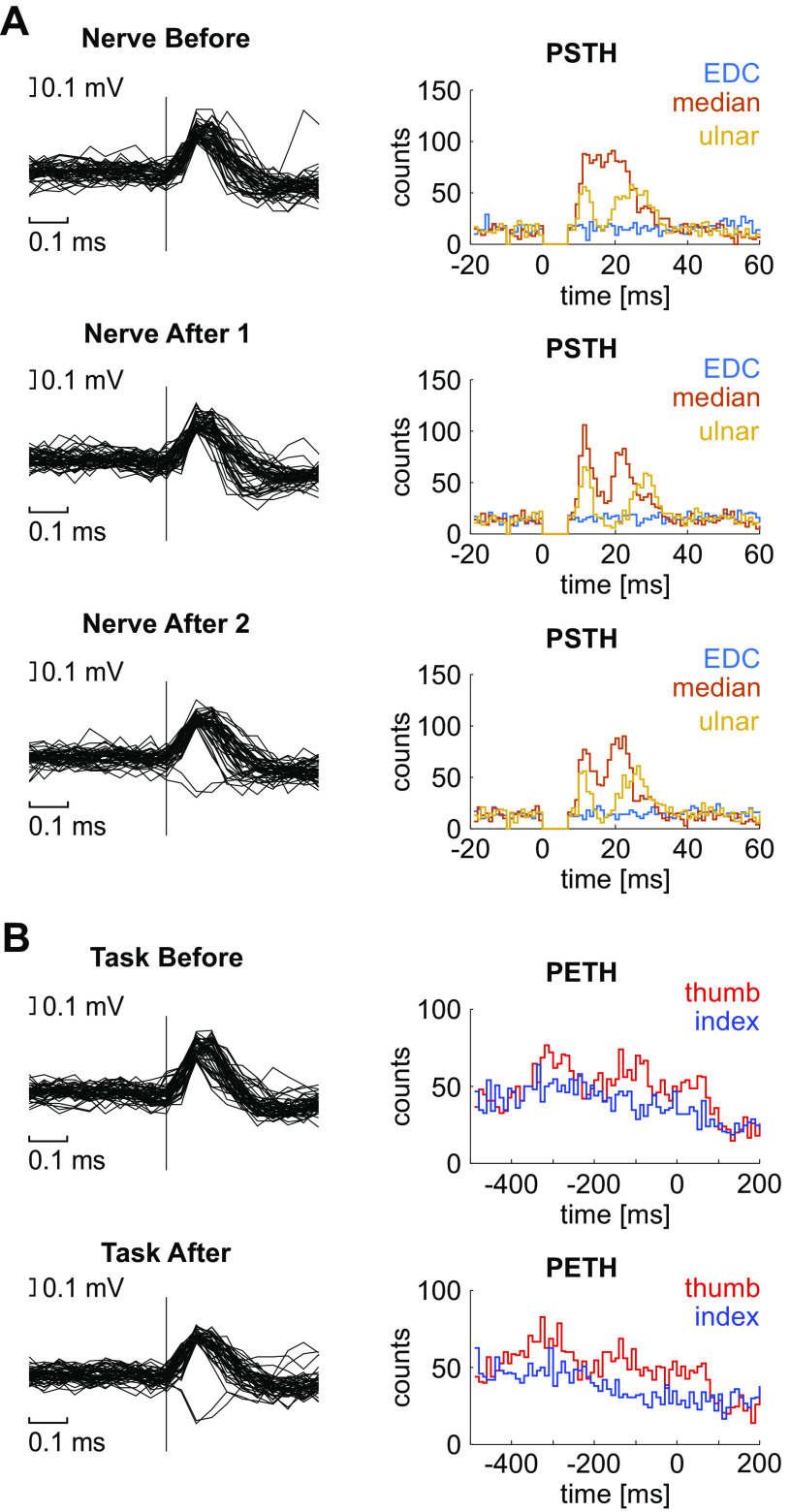
Example of spike waveforms and peristimulus time histograms (PSTHs) across a single session. *A*: aligned spike waveforms and PSTHs of the same MUA across one session during individual nerve stimulation assessment before (*top*), immediately after (*middle*), and approximately 40 min after (*bottom*) asynchronous stimulation. The ordinate of the PSTHs shows number of spikes per bin. *B*: same MUA during behavioral task before (*top*) and after (*bottom*) asynchronous stimulation, for trials where thumb or index finger movement was instructed. Spike waveforms were aligned to the completion of successful trials. EDC, extensor digitorum communis; MUA, multiunit activity; PETH, perievent time histogram.

In total, 195 MUAs (86 from monkey S, 109 from monkey U) and 11 stable PTNs (two from monkey S, nine from monkey U) were included in the analysis. At 63 of these sites, a train of intracortical microstimulation elicited wrist or digit movements with thresholds ≤40 μA. These penetrations were intermixed with the remainder of sites, which either did not produce low-threshold distal extremity movements or were not tested for technical reasons, indicating that recordings came from the hand representation of M1. Figure 4 shows example waveforms and PSTH profiles for one example MUA, for each assessment before and after the intervention. The illustrated MUA showed a short-latency response to both ulnar and median nerve stimulation but no response to stimulation of the EDC electrode. There was a modulation with task performance, which was clearly different for thumb and index finger instructed trials.

### Changes in Neural Responses to Peripheral Stimuli after Paired Nerve Stimulation

**Figure 5.**
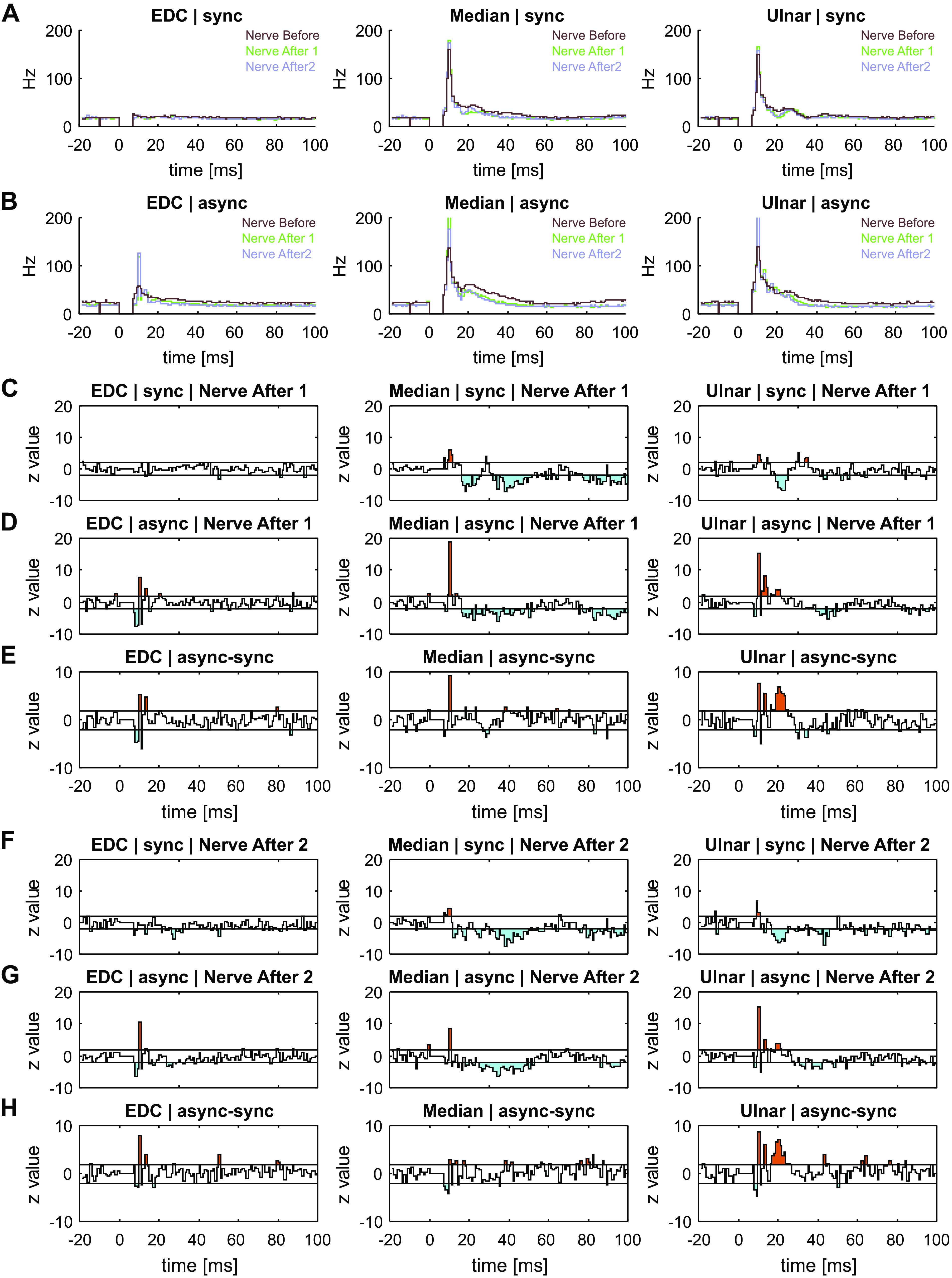
MUA after synchronous and asynchronous stimulation for EDC (*left*), median (*middle*), and ulnar (*right*) stimulation. *A*: average PSTH across all MUAs (*n* = 107), before (brown), immediately after (green), and 40 min after (purple) synchronous stimulation. *B*: average PSTH across all MUAs (*N* = 88), before (brown), immediately after (green), and 40 min after (purple) asynchronous stimulation. *C*: population *Z*-scores immediately after (*nerve after 1*) synchronous stimulation. *D*: population *Z*-scores immediately after asynchronous stimulation. *E*: difference between population *Z*-scores immediately after (*nerve after 1*) synchronous and asynchronous stimulation. *F*: population *Z*-scores after 40 min (*nerve after 2*) of synchronous stimulation. *G*: population *Z*-scores after 40 min (*nerve after 2*) of asynchronous stimulation. *H*: difference between population *Z*-scores after 40 min of synchronous and asynchronous stimulation. In *C*–*H*, bins greater (orange) or less (cyan) than the 95% confidence intervals (horizontal lines) are shaded. async, asynchronous stimulation; EDC, extensor digitorum communis; MUA, multiunit activity; PSTH, peristimulus time histogram; sync, synchronous stimulation.

Figure 5 presents data on how neurons responded to peripheral stimulation and how this was changed by the period of paired nerve stimuli (comparison of *nerve before* with *nerve after 1* and *nerve after 2* as illustrated in Fig. 2*A*).

Figure 5*A* shows the average PSTH profiles across all MUA during the stimulation assessments before and after synchronous nerve stimulation. Figure 5*B* shows the average PSTH profiles before and after asynchronous stimulation. There were no consistent differences in the type of cell firing seen in baseline responses (*nerve before*) before synchronous or asynchronous nerve stimulation (*P* = 0.015 for EDC, not significant after correcting for multiple comparisons, *P* = 0.102 for median, *P* = 0.109 for ulnar).

The remaining plots in Fig. 5 show *Z* scores, which indicate how the neural response to peripheral stimuli changed after paired stimulation of the ulnar and median nerve (*nerve before* vs. *nerve after 1*). If there were no consistent changes in cell firing after the paired stimulation compared to before, the *Z* score should have a mean of zero, and standard deviation of one. A reduction in firing rate after stimulation is shown by *Z* < 0, whereas *Z* > 0 if the firing rate increased. After synchronous stimuli (Fig. 5*C*), there was minimal change in the response to EDC; this might be expected, as these afferents had not been involved in the stimulus pairing and therefore served as a control.

The earliest response to both median and ulnar nerve was increased after stimulus pairing. This was followed by a decrease in long-latency responses, which was more pronounced and of longer duration in response to the median compared with the ulnar nerve (Fig. 5*C*).

For asynchronous stimulation, surprisingly there was an early reduction and increase in the response to EDC stimulation (Fig. 5*D*). There was also a markedly greater increase in response to both median and ulnar nerve stimulation (Fig. 5*D*), and the later decrease in firing was not as pronounced as for the synchronous stimulation.

Figure 5*E* presents normalized differences of these two sets of *Z* score time courses, allowing a comparison of the effects of synchronous and asynchronous paired stimulation The difference has been renormalized by dividing by $\sqrt{2}$ to ensure that these plots should have zero mean and unit variance under the null hypothesis that the changes following synchronous and asynchronous stimulation are the same. This confirmed that asynchronous stimulation produced a significantly larger increase in the short-latency response to EDC than synchronous stimulation although this may be related to the differences in baseline responses seen in these two data set. The increase in the very earliest part of the response to median and ulnar nerve stimuli was significantly greater following asynchronous versus synchronous stimulation.

Figure 5, *F–H*, shows the results of a similar analysis but now comparing responses to peripheral stimuli recorded before and around 40 min after the end of the paired nerve stimulation (*nerve before* vs. *nerve after 2*). Very similar effects are apparent as for the measurements taken immediately after the paired stimulation (Fig. 5, *C*–*E*). This indicates that the plastic changes in neural responses to peripheral stimuli were long-lasting.

### Changes in Task-Related Neural Activity after Paired Nerve Stimulation

**Figure 6.**
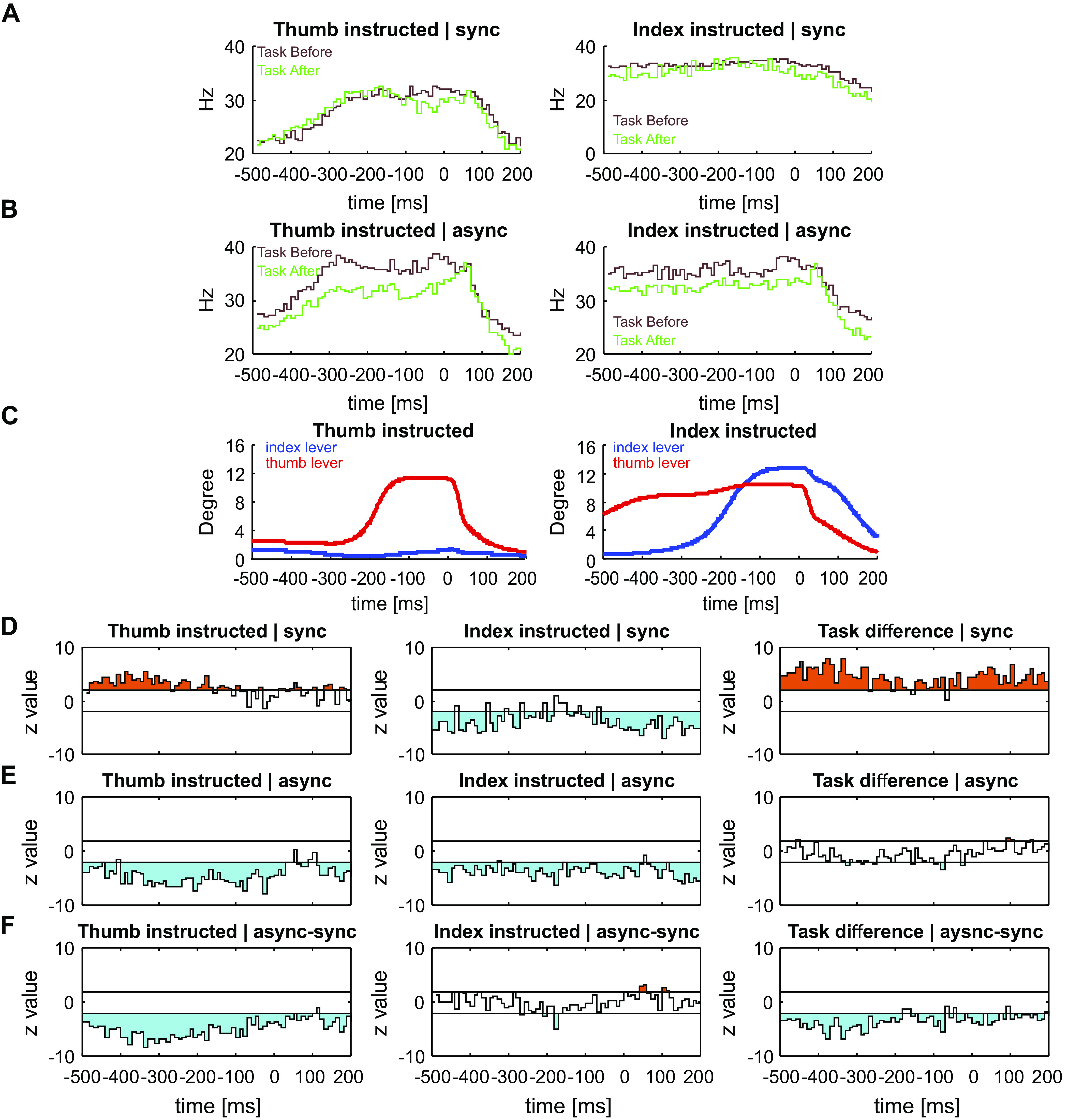
MUA activity during task performance for thumb- (*left*) and index finger (*right*)-instructed trials*. A*: average PETH across all MUAs (*n* = 107), before (brown) and after (green) synchronous stimulation. *B*: average PETH across all MUAs (*n* = 88), before (brown) and after (green) asynchronous stimulation. *C*: average lever positions for the thumb (red) and index (blue) lever, before the stimulation. *D*: population *Z*-scores comparing multiunit differences during task performance after synchronous stimulation. The column on the right shows the difference between the two tasks (thumb-index instructed). *E*: population *Z*-scores comparing MUA differences during task performance after asynchronous stimulation. The column on the right shows the difference between the two tasks (thumb-index instructed). *F*: difference between population *Z*-scores after synchronous and asynchronous stimulation. The column on the right shows the difference between the two tasks (thumb-index instructed). In *D*–*F*, bins greater (orange) or less (cyan) than the 95% confidence intervals (horizontal lines) are shaded. async, asynchronous stimulation; MUA, multiunit activity; PETH, perievent time histogram; sync, synchronous stimulation.

Figure 6, *A* and *B*, shows average PETH profiles across all MUA during task assessment before and after synchronous (Fig. 6*A*) and asynchronous (Fig. 6*B*) stimulation (comparison of *task before* and *task after* as illustrated in Fig. 2*A*). There was no clear difference between baseline (*task before*) PETH profiles for synchronous and asynchronous nerve stimulation, and MUA firing rates were not significantly different at baseline (*P* = 0.167 for thumb-instructed trials, *P* = 0.607 for index-instructed trials) although MUA firing rates in the sample during experiments with synchronous stimulation appeared slightly higher than for asynchronous stimulation. The corresponding finger movements for the baseline task assessment (*task before*) are illustrated as the average lever positions across all sessions, plotted in Fig. 6*C*.

To evaluate whether there were consistent changes in how cells fired after the period of paired nerve stimulation, we computed *Z* scores. This was similar as for the nerve stimulation data described above, except that no baseline period was used (see methods). Figure 6*D* shows the results of this analysis after synchronous nerve stimulation, combined over 99 recordings of MUA. From around 500 ms before to 200 ms after the end of a trial which instructed thumb movement, population activity was significantly increased after the paired nerve stimulation (orange shaded area indicates |*Z*|>1.96, corresponding to *P* < 0.05). By contrast, activity was significantly decreased during this period for trials where index finger movement was instructed. The right column in Fig. 6*D* shows the difference between the two tasks (thumb-instructed trial − index-instructed trial), renormalized by dividing by $\sqrt{2}$ so that it should lie within |*Z*|<1.96 if the same change was seen for each type of trial. In fact, this difference was consistently positive, indicating a significant difference between the effects on index- and thumb-instructed trials.

Figure 6*E* presents a similar analysis for recording sessions where asynchronous nerve stimulation was delivered, combined over 90 recordings of MUA. For trials where index finger movement was instructed, broadly similar changes occurred as following synchronous stimulation, with an overall decrease in MUA rate. Interestingly, activity was also significantly decreased for trials where thumb movement was instructed. There were barely any differences between the two tasks after asynchronous stimulation (right column, Fig. 6*E*).

Figure 6*F* presents the difference of the traces shown in Fig. 6, *D* and *E* (similar to the analysis presented in Fig. 5*E* for peripheral stimuli responses); this reveals whether there were any significant differences between the two types of stimulation. There was a clear difference for thumb-instructed trials, with the two types of paired stimulation inducing opposite effects. For index-instructed trials, there was only a brief period around 200 ms before the end of a trial where the effects of synchronous and asynchronous stimulation differed; there was a significantly greater reduction in MUA rate following asynchronous stimulation.

### Neural Activity Can Classify Stimulus or Trial Type at Above Chance Levels

The analysis of the previous section revealed a clear modulation in neural activity at the population level by peripheral stimuli and during task performance. Furthermore, there were some consistent changes in this averaged activity after paired stimulation. We next investigated how effectively individual MUA recordings could encode what type of movement was being made, or what peripheral stimulus was being given. This aimed to measure whether coding was altered (enhanced or degraded) after paired stimulation.

**Figure 7.**
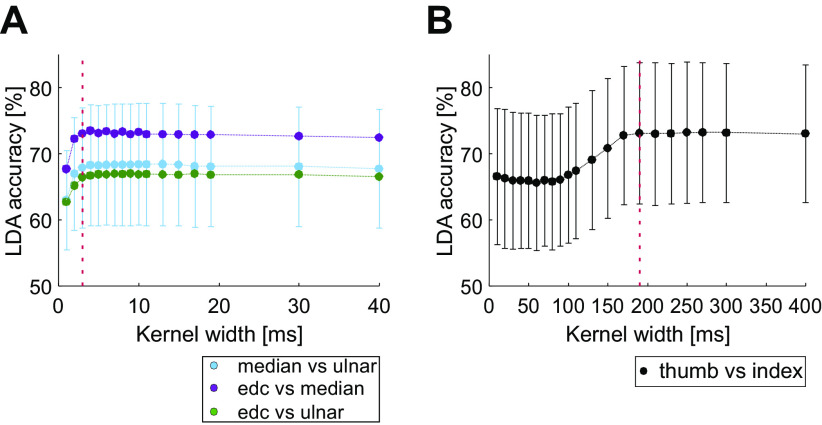
Relationship of linear discriminant analysis (LDA) accuracy and chosen width of the Gaussian kernel for instantaneous rate estimation. *A*: for classification of responses to nerve stimulation, (*B*) for the behavioral task. Results are shown as the average across all available MUA recordings before the paired nerve stimulation; error bars show standard deviation. Error bars are shown only for median vs. ulnar classification in *A* for clarity; error bars for the other classifications were similar in size. Vertical dotted lines show the kernel widths chosen for subsequent analysis (*A*, 190 ms; *B*, 3 ms). edc, Extensor digitorum communis; MUA, multiunit activity.

To investigate this question, we exploited linear discriminant analysis (LDA). This used a single trial estimate of the MUA firing rate as the input to a classifier, whose output was a prediction of the type of that trial (e.g., index finger- vs. thumb-instructed digit, or ulnar versus median nerve stimulation). One key parameter in LDA is the degree of smoothing of the single trial neural data, which is controlled by the width parameter of the Gaussian kernel. Too narrow a kernel width will produce an instantaneous firing rate that simply replicates the timing of the individual spikes; too wide a kernel will oversmooth and could lose differences between conditions at short time scales. We accordingly checked how the accuracy of LDA classification depended on kernel width. The results are shown in Fig. 7. In all cases, LDA accuracy was significantly higher than the 50% expected if classification performed only at chance levels (*P* < 0.0001, Monte Carlo analysis). For the responses to peripheral stimulation (Fig. 7*A*), LDA accuracy rose rapidly for kernel widths from 1 to 3 ms, but then reached a plateau for higher values; this reflects the sharp and narrow peaks in the PSTH which often followed peripheral stimulation (see examples in Fig. 4 and Fig. 5). For task-related modulation, wider kernels were required for optimal performance, with a plateau reached after a width of 190 ms (Fig. 7*B*). We accordingly used 3-ms kernels for responses to peripheral stimuli, and 190 ms for behavioral task modulation, in all subsequent analysis (marked by the dotted lines in Fig. 7).

### Changes in the Neural Coding of Peripheral Stimuli after Paired Stimulation

**Figure 8.**
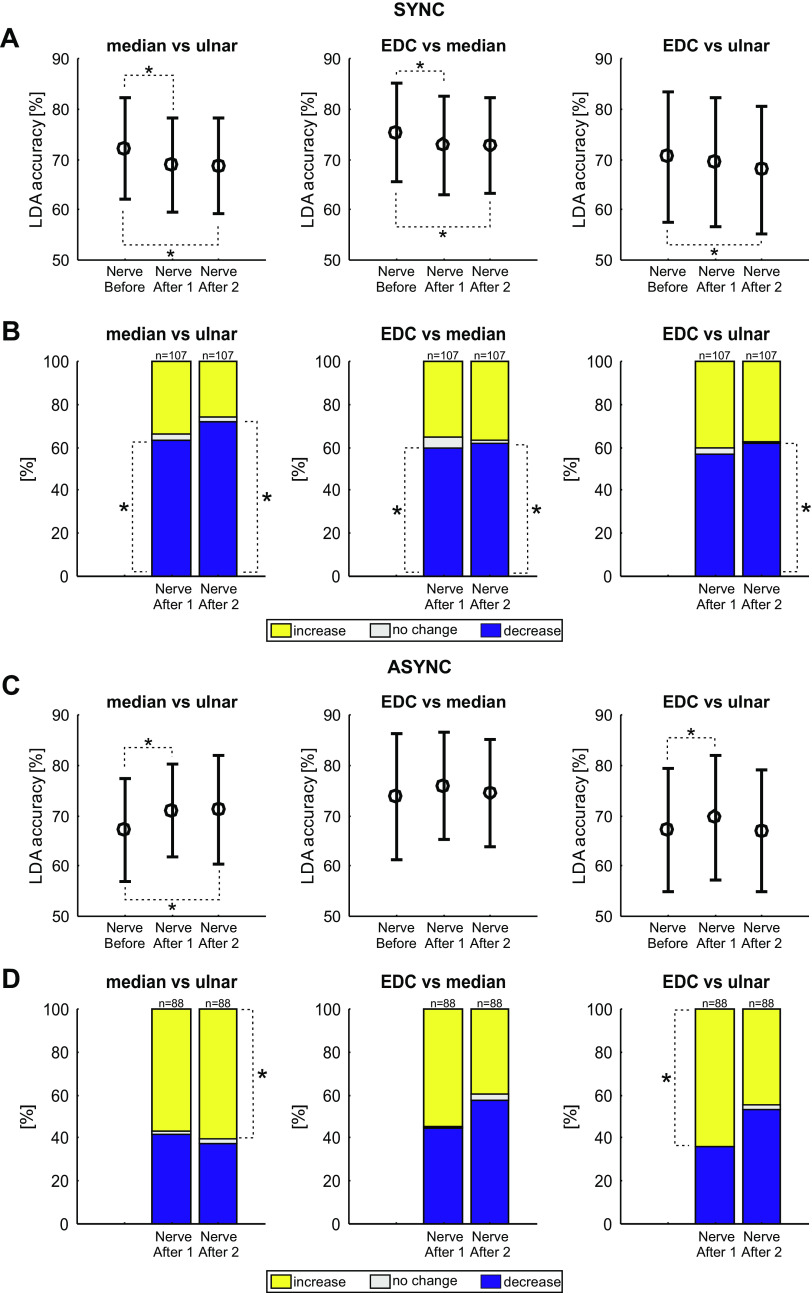
Linear discriminant analysis (LDA) accuracies for multiunit activities (MUAs) for each pair of nerves during individual nerve stimulation assessments. *A*: mean LDA accuracies for MUA (*n* = 107) before, immediately after, and approximately 40 min after (*nerve before*, *nerve after 1*, and *nerve after 2*, respectively) synchronous stimulation. Significant differences between the timepoints are indicated with an asterisk (*P* < 0.05, Wilcoxon signed-rank test, adjusted for multiple comparisons with the Benjamini–Hochberg procedure). Error bars indicate standard deviations. *B*: the percentage of units that showed an increase (yellow), no change (white), or decrease (blue) in number of correctly predicted trials after compared with before the intervention. Asterisks indicate proportions significantly different than expected by chance (*P* < 0.05, Monte Carlo test, adjusted for multiple comparisons with the Benjamini–Hochberg procedure). *C* and *D*: as *A* and *B*, but for asynchronous paired stimulation (*n* = 88 MUAs). async, Asynchronous stimulation; EDC, extensor digitorum communis; sync, synchronous stimulation.

Figure 8 shows how the average LDA classification accuracy of the neural responses to peripheral stimulation changed after paired nerve stimulation (comparison of *nerve before* with *nerve after 1* and *nerve after 2* as illustrated in Fig. 2*A*). Each plot presents results for a pairwise classification: median versus ulnar, EDC versus median, and EDC versus ulnar. Results are shown separately for the synchronous (Fig. 8*A*) and asynchronous (Fig. 8*C*) paired stimulation. In all cases the mean LDA accuracy was above 50% (Monte Carlo test, *P* < 0.0001), indicating that average classification rates were above chance for all comparisons. The LDA accuracy was reduced by synchronous stimulation for comparisons of median responses with both ulnar and EDC responses (Fig. 8*A*). This effect was long-lasting, being present in both the assessment immediately after (*nerve after 1*; median vs. ulnar: *P* = 8.10 × 10^−7^, median vs. EDC: *P* = 0.004) the paired stimulation, and 40 min later (*nerve after 2*; median vs. ulnar: *P* = 1.03 × 10^−6^, median vs. EDC: *P* = 0.006). LDA accuracy was also reduced by synchronous stimulation for comparison of EDC and ulnar responses (EDC vs. ulnar: *P* = 0.006), but only for the assessment around 40 min after paired stimulation (*nerve after 2*).

By contrast, LDA accuracy was increased by asynchronous stimulation (Fig. 8*C*) for median versus ulnar stimuli in the assessment immediately after paired stimulation (*P* = 0.016, *nerve after 1*) and 40 min later (*P* = 0.017, *nerve after 2*). There was also a significant change in LDA accuracy after asynchronous stimulation for EDC versus ulnar stimuli (*P* = 0.008), immediately after the asynchronous intervention (*nerve after 1*).

The plots of Fig. 8, *A* and *C*, indicate the average changes seen in LDA accuracy across all recorded MUA. As a complementary analysis, Fig. 8, *B* and *D*, indicates the proportion of MUA where classification accuracy increased, decreased, or stayed the same. For synchronous stimulation, the results of this alternative analysis agreed closely with that using averaged LDA accuracy. Both immediately after, and 40 min after synchronous stimulation, more MUAs decreased their classification accuracy than expected by chance for the median versus ulnar (*P* = 0.003 and *P* < 0.0001, *nerve before* compared with *nerve after 1* and *nerve after 2*, respectively) and median versus EDC (*P* = 0.007 and *P* = 0.015) comparisons (Fig. 8*B*, *significance calculated by a Monte Carlo procedure). There was also a significant proportion of MUAs decreasing their classification accuracy for the EDC versus ulnar comparisons 40 min after synchronous stimulation (*P* = 0.020).

For asynchronous stimulation, the proportion of MUAs increasing LDA accuracy was significant different for the median versus ulnar comparison (*P* = 0.136 and *P* = 0.020, for *nerve before* vs. *nerve after 1* and *nerve before* vs. *nerve after 2*, respectively) and the EDC versus ulnar comparison (*P* = 0.008 and *P* = 0.385).

These results indicate that after a period of synchronous stimulation, the neural discharge following peripheral stimuli became less distinguishable—an ideal observer would be less able to tell the different types of stimulation apart. By contrast, after a period of asynchronous stimuli, the neural discharge was in some cases more separated, so that an ideal observer could better determine which stimulus had occurred.

**Figure 9.**
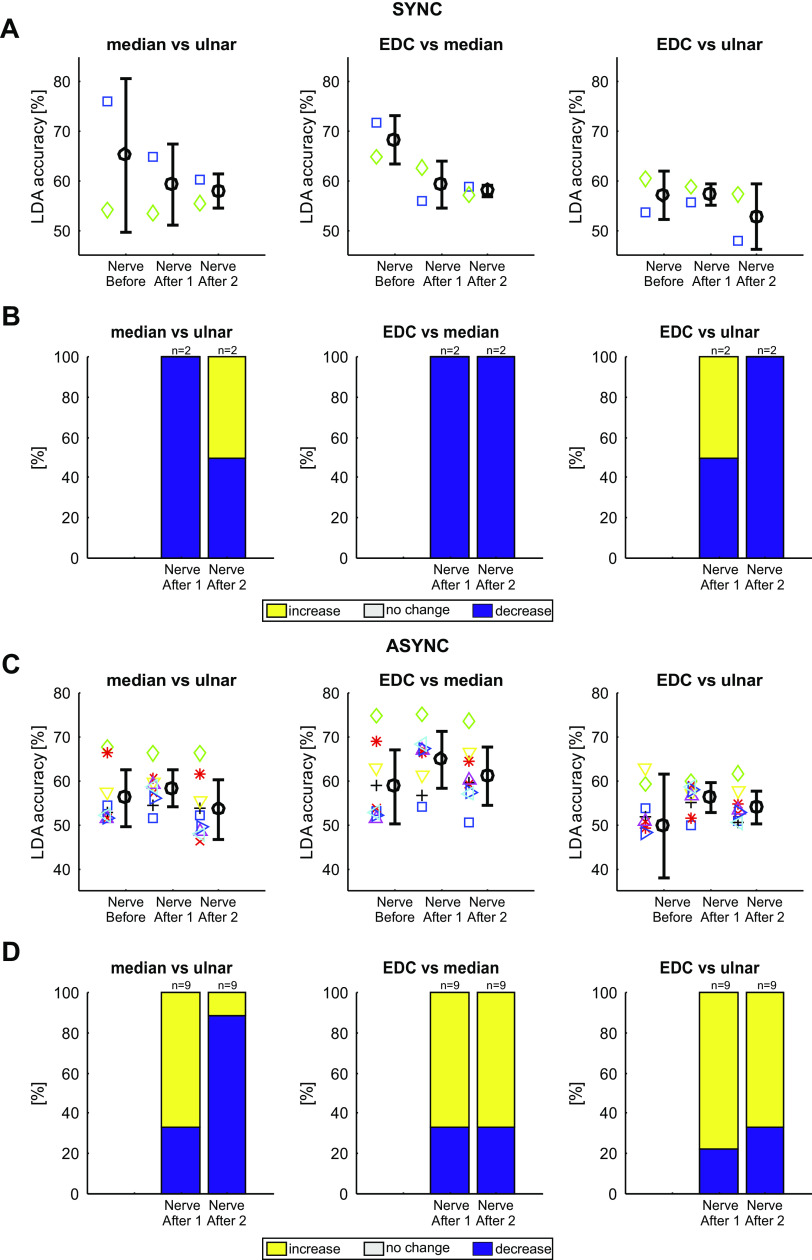
Linear discriminant analysis (LDA) accuracies for pyramidal tract neurons (PTNs) for each pair of nerves during individual nerve stimulation assessments. *A*: mean LDA accuracies for PTNs (*n* = 2) before, immediately after, and approximately 40 min after (*nerve before*, *nerve after 1*, and *nerve after 2*) synchronous stimulation. Mean and standard deviations are shown in black; individual PTNs are represented as differently colored symbols. *B*: the percentage of units that showed an increase (yellow), no change (white), or decrease (blue) in number of correctly predicted trials compared with before the intervention. *C* and *D*: as *A* and *B*, but for asynchronous paired nerve stimulation (*n* = 9 PTNs). async, Asynchronous stimulation; EDC, extensor digitorum communis; sync, synchronous stimulation.

We were able to maintain stable recordings from only a small number of identified PTNs throughout our lengthy protocol; results from these are presented in Fig. 9, in a similar format to the analysis of MUA shown in Fig. 8. The small number of cells available limited the conclusions that could be drawn. However, it is notable that of the 12 measurements available after synchronous stimulation, 10 of 12 showed a decrease in classification accuracy, broadly similar to the predominant decreases seen in the more extensive MUA data set. By comparison, after asynchronous stimulation, 32 of the 54 available measurements showed an increase in accuracy, similar to the preponderance of increases seen in the MUA.

### Changes in the Neural Coding of Behavioral Task after Paired Stimulation

**Figure 10.**
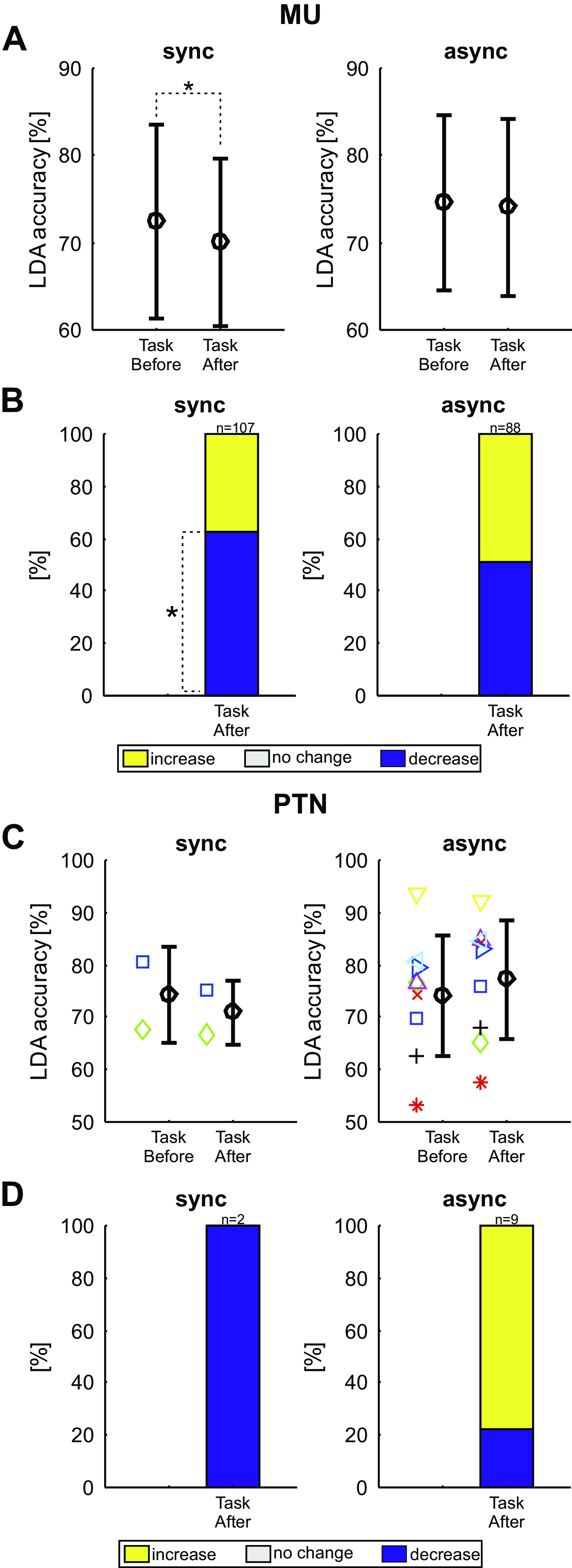
Linear discriminant analysis (LDA) accuracies during the behavioral task assessment. *A*: mean LDA accuracies for multiunits (MUs) before and approximately 15 min after synchronous (*left*, *n* = 107 MUs) and asynchronous (*right*, *n* = 88 MUs) stimulation. Error bars indicate standard deviations. *B*: the percentage of units that showed an increase (yellow), no change (white), or decrease (blue) in number of correctly predicted trials compared with before the intervention. Asterisks indicate significant portions (Monte Carlo analysis, *P* < 0.05, adjusted for multiple comparisons with the Benjamini–Hochberg procedure). *C*: as for *A*, but identified for pyramidal tract neurons (PTNs). Mean and standard deviations are shown in black; individual PTNs are represented as differently colored symbols. *D*: as for *B*, but for PTNs. async, Asynchronous stimulation; sync, synchronous stimulation.

Figure 10 presents the results using an LDA classifier to predict if a trial of the behavioral task involved instructed index finger or thumb movements, and how this changed with paired stimulation (comparison of *task before* with *task after* as illustrated in Fig. 2*A*). Once again, the average LDA accuracy across all MUAs was significantly above the chance level of 50% in all cases (Monte Carlo test, *P* < 0.0001). The mean LDA accuracy decreased slightly but significantly (*P* = 0.032) after the synchronous paired stimulation (Fig. 10*A*, *left*). There was no change in mean LDA accuracy after the asynchronous paired stimulation (*P* = 0.529, Fig. 10*A*, *right*). Significantly more MUAs showed a decrease in LDA accuracy than an increase for the synchronous stimulation (*P* = 0.019, Monte Carlo test, Fig. 10*B*, *left*). By contrast, similar proportions of MUAs showed increases or decreases in LDA accuracy after asynchronous paired stimulation (*P* = 0.756, Fig. 10*B*, *right*). These results indicate that following a period of synchronous paired stimulation, neural activity in M1 represented the difference between the two finger movements less effectively—just as M1 activity for different peripheral stimuli became more similar. However, the distinction between the two movements was unchanged after a period of asynchronous paired stimulation.

Fig. 10, *C* and *D*, presents data for the available identified PTNs, in a similar format to Fig. 9. Once again, the small number of cells available precludes any definitive conclusions, but the changes seen are consistent with those seen in the more extensive MUA data set.

### Controlling for Possible Effect of Recording Instability on LDA Accuracy Changes

The main results presented in this report involve MUA. Unlike well isolated single units, it is possible that MUA recordings changed during a recording session due to uncontrolled tissue movements relative to the electrode. This could lead to the loss (or gain) of a unit, which provided good classification accuracy between conditions; LDA accuracy would then change, simply as an artifact of recording instability. To check for this, we measured the change in firing rate across different recording epochs as a marker of recording instability and calculated the correlation with change in LDA accuracy. Of the seven possible correlations (behavioral task and three pairwise comparisons of peripheral stimuli each at two timepoints after paired stimulation), no correlation coefficient was significantly different from zero after adjusting for multiple comparisons. Also, all correlation coefficients were small (*r*^2^ ranged from 0.0042 to 0.0216), suggesting that changes in rate did not contribute to changes in LDA accuracy.

## DISCUSSION

In this report, we show that long-term plastic changes can be induced in M1 activity following a period of paired nerve stimulation. Although other approaches are capable of modulating net cortical output, this type of stimulation is particularly interesting because it was able to change the selectivity of cortical encoding. Paired nerve stimulation can be easily applied in humans noninvasively for long periods, suggesting that it may prove to be a useful tool in the armory of neurostimulation approaches to aid functional recovery in patients.

### Technical Considerations

The long duration of our experiments (around two and a half hours) made it difficult to track and retain recordings from the same single unit throughout. Because many recordings failed to meet our usual high standards for single units, we instead decided to quantify neural activity using MUA. Many other previous studies have relied on MUA, and in some circumstances, it can have advantages over single units (53). However, it could raise problems of interpretation: the loss of one single unit from the recording, or the appearance of another, could alter response rates and coding efficacy measured by *Z* score analysis and LDA, respectively. We cannot rule this out for any individual recording; however, several features of our data set suggest that artifacts alone could not explain the findings. Correlations between measured changes after paired stimulation and changes in MUA firing rate were small. Changes were much larger for the responses to ulnar and median nerve stimulation than for EDC (Fig. 5). This is expected if changes reflect plasticity, since it was the ulnar and median nerves which were paired, whereas artifactual changes due to recording instability should influence all response equally. For synchronous stimulation there was an average increase in responses to thumb-instructed trials and decrease in index finger-instructed trials (Fig. 6*D*); if changes reflected random loss or gain of units from the recording, then the different types of behavioral trial should on average be affected equally. Additionally, opposite changes in average LDA accuracy were seen after synchronous and asynchronous stimulation (Fig. 8, *A* and *C*); these should be equally affected by instability. Furthermore, although marked changes were seen both in average activity and LDA accuracy, between baseline measurements and those immediately after the period of paired stimulation (Fig. 5, *C* and *D*; Fig. 8, *A* and *C*), there was remarkably little further change in the measurements made around 40 min later (Fig. 5, *F* and *G*; Fig. 8, *A* and *C*). We would expect recording instability to change progressively with time, whereas here changes seemed firmly associated with the paired stimulation. Finally, the small number of cleanly identified and tracked single PTNs which were available behaved in a similar way to the averaged MUA activity. We therefore conclude that our results likely reflect genuine plastic changes in cortical activity caused by the paired stimulation.

To measure how well neural activity could represent and distinguish different stimuli, or performance of different behaviors, we measured the accuracy of decoding using LDA. It is possible that a more sophisticated approach, using for example nonlinear methods, could have achieved higher accuracy. However, our interest here was to test whether coding was altered after paired stimulation, not to make absolute estimates of the coding quality. It is unlikely that the changes observed following paired stimulation would be materially altered by using a different decoding strategy, even if better accuracy could be achieved from a given MUA recording.

The monkeys in this study performed two different types of trial, but it must be noted that these were not simply inverses of each other. For thumb-instructed trials, the thumb moved more than the index finger; by contrast, for index-instructed trials, both digits moved rather similarly (Fig. 1). This seemed to reflect an intrinsic behavioral limitation of our monkeys; despite considerable training, neither animal was able to produce a more selective index finger abduction. This is in contrast to flexion-extension movements of the digits, where a high level of individuation can be achieved by trained monkeys (54). However, even then while thumb flexion is associated on average with no index finger movement, index finger flexion consistently produces a small thumb flexion (see Table 2 in Ref. 54). For the purposes of this study, we required only that the monkey produced consistent hand movements (for assessment of net activity changes) and performed two clearly different hand movements (for assessment of coding changes using LDA). The behavioral performance was sufficient for these purposes.

### Long-Term Changes in Central Circuits after Peripheral Stimulation

After 1 h of paired stimulation of the ulnar and median nerves, we saw consistent changes in neural activity in M1. The very earliest part of the response to both ulnar and median nerve inputs, starting around 7 ms, was increased after both synchronous and asynchronous stimulation (Fig. 5, *C* and *D*). The later component, up to 30 ms, could be affected differently depending on the type of stimulus pairing. For the ulnar nerve responses, this component was increased after asynchronous, but decreased after synchronous stimulation. Both types of paired stimulation decreased responses to the median nerve. This is likely to reflect long-term adaptation following the period of constant stimulation. Similar effects occur in the visual cortex, where they can produce motion aftereffects (55), and in the auditory system (56). However, it should be noted that the effects were relatively long-lasting in this study, persisting between the first and second measurements of the neural responses to nerve stimulation, which were separated by around 40 min. This is a longer timescale than often reported for adaptation in sensory cortices.

Changes in responses to EDC stimulation were much smaller than to ulnar or median nerves. This might be expected, as the radial nerve afferents activated by EDC stimulation were not activated during the 1-h period of paired nerve stimulation. The long-term plastic changes therefore seem to obey the principle of specificity. Schabrun and Ridding (22) also reported smaller changes in response to TMS in the muscles not included in the paired stimulation.

### Synchronous versus Asynchronous Stimulation

Human experiments have described two distinct types of plastic change following a period of paired nerve stimulation. First, motor output [assessed by the size of motor-evoked potentials (MEPs) after magnetic stimulation of M1] can increase. This occurs only when paired stimuli are delivered synchronously (19, 21, 22, 24, 26); asynchronous stimulation does not alter MEP amplitude (21, 22). The changes that we observed in M1 activity during task performance were somewhat consistent with these cortical excitability changes in humans. Neural activity during task performance was increased overall by synchronous stimulation, and decreased by asynchronous stimulation (Fig. 6), although the precise details of this modulation depended on the task. By contrast, synchronous and asynchronous stimulation had similar effects on the responses to peripheral nerve stimulation, with the early response component enhanced, and late component reduced (Fig. 5, *C, D, F,* and *G*). Overall, asynchronous stimulation increased responses to nerve stimulation more than synchronous (Fig. 5, *E* and *H*), which is the opposite of what we might expect based on the human studies. It is important to realize however that changes in cortical excitability using TMS cannot necessarily be equated with changes in cortical activity. It is known, for example, that TMS produces a greater descending volley during the phases of a precision grip task when cortical activity is least (57). Caution is especially required here, since the TMS measures in humans were made at rest, whereas we studied responses during task performance or to nerve stimulation. Our data reveal that paired nerve stimuli have complex effects on net cortical activity, which are not apparent from non-invasive measures in humans.

The second type of change seen in human subjects was observed in patients with focal task-specific dystonia. Maps of the representation of muscles in M1 were constructed using the responses to TMS delivered at regularly spaced sites; asynchronous paired stimulation increased the separation in the map of muscles supplied by the stimulated nerves (32, 33). Our results with LDA were all consistent with this effect. Neural responses to ulnar and median nerve stimulation became less distinguishable after synchronous, and more distinguishable after asynchronous paired stimulation (Fig. 8). The modulation of neural activity in the index and thumb movement tasks was less separable after synchronous stimulation; asynchronous stimulation had no effect (Fig. 10). These changes in neural activity were sufficient to produce behavioral changes: measures of task performance decreased after synchronous but increased after asynchronous stimulation.

We recently characterized muscle recruitment by different cortical and subcortical centers and suggested that motor cortex makes a distinctive contribution to precise patterning of multiple muscles (58). If the ability of M1 neural activity to code inputs and movements selectively is impaired by synchronous stimulation, it may not be possible for other centers to compensate. On this view, any benefit (e.g., to patients after damage to the motor system) from enhanced cortical output generated by synchronous stimulation might have to be offset against the resulting less effective selective cortical activation and the consequent reduction in behavioral performance on highly fractionated movements. A better strategy to increase overall output might be to target subcortical pathways using alternative stimulation strategies (27). This could yield increases in strength (59) without sacrificing cortical selectivity. On the other hand, the ability of asynchronous paired nerve stimuli to induce long-term enhancements in cortical selectivity might provide a unique way to improve fractionation of motor output. Further development of these different stimulation approaches could allow individually tailored treatments to target strength and individuation, the two major components of functional recovery such as after stroke (60).

### Anatomical Site and Mechanism Underlying Observed Changes

Somatosensory input reaches the hand representation of M1 via the dorsal columns, cuneate nucleus, medial lemniscus, and ventroposterior lateral (VPL) nucleus of the thalamus. Some inputs come direct from VPL to M1 (61); others are routed via the somatosensory cortex (62). After paired nerve stimulation, we observed changes in even the earliest neural responses to peripheral stimuli. Although this could result from changed synaptic efficacy within the cortex, we cannot rule out changes in downstream circuits. We know that the cuneate nucleus contains sophisticated local circuitry (63), which can modulate the frequency content of information transmission (64). Little is known about plasticity in the cuneate following stimulation although after spinal cord lesions there are substantial plastic changes and reorganization (65, 66). However, it seems likely that some changes do also occur in the cortex itself after paired stimulation, given the altered behavioral performance and changes in neural firing with task performance that we observed. Paired stimulation also produced decreases in the long-latency activation of neural activity following peripheral stimulation; this is likely to be mediated by local cortical circuits.

The mechanism underlying long term plastic changes is often assumed to be spike-timing dependent plasticity (STDP; 67). A downstream neuron receiving convergent input from two sources will be more likely to be activated if both sources are stimulated synchronously; the activation of a postsynaptic spike just after presynaptic input could then lead to synaptic facilitation via STDP (28). However, in this study, we observed an overall decrease in responses to median and ulnar nerve stimulation after synchronous stimulation; this is the opposite to what we would expect from STDP. A facilitation of the population responses *was* seen after asynchronous stimulation, but there the interstimulus intervals were too long to invoke STDP mechanisms. Recent reports have suggested that plasticity may also occur with wider spaced stimuli, reflecting presumably other underlying mechanisms (28, 68, 69). We assume that similar processes are also at work here, alongside STDP.

## GRANTS

This work was funded by Wellcome Trust (WT092995MF). M. Germann was supported by the Medical Research Council (MR/P023967/1) and the International Spinal Research Trust (NRB118).

## DISCLOSURES

No conflicts of interest, financial or otherwise, are declared by the authors.

## AUTHOR CONTRIBUTIONS

B.H. and S.N.B. conceived and designed research; B.H. and S.N.B. performed experiments; B.H. and M.G. analyzed data; B.H., M.G., and S.N.B. interpreted results of experiments; M.G. prepared figures; M.G. and S.N.B. drafted manuscript; M.G. and S.N.B. edited and revised manuscript; B.H., M.G., and S.N.B. approved final version of manuscript.
